# Long-term exposure to heavy physical work, disability pension due to musculoskeletal disorders and all-cause mortality: 20-year follow-up—introducing Helsinki Health Study job exposure matrix

**DOI:** 10.1007/s00420-018-1393-5

**Published:** 2018-12-03

**Authors:** Jenni Ervasti, Olli Pietiläinen, Ossi Rahkonen, Eero Lahelma, Anne Kouvonen, Tea Lallukka, Minna Mänty

**Affiliations:** 1 Department of Public Health, University of Helsinki, Helsinki, Finland; 2 Finnish Institute of Occupational Health, PB 18, 00032 Helsinki, Finland; 3 Faculty of Social Sciences, University of Helsinki, Helsinki, Finland; 4 SWPS University of Social Sciences and Humanities in Wroclaw, Wroclaw, Poland; 5 Administrative Data Research Centre-Northern Ireland (ADRC-NI), Queen’s University Belfast, Belfast, UK; 6 Laurea University of Applied Sciences, Vantaa, Finland

**Keywords:** Disability pension, Job exposure matrix, Mortality, Physical job demands

## Abstract

**Purpose:**

We developed a job exposure matrix (JEM) to study the association between long-term exposure to heavy physical effort or heavy lifting and carrying at work with disability pension due to musculoskeletal disorders and premature all-cause mortality.

**Methods:**

Exposure to heavy physical effort at work during 1996–2005 was estimated with JEM developed for this study population, where the exposure was based on occupational titles of the participants. We included all employees of the City of Helsinki, Finland, who had annual data of exposure for 8–10 years (1996–2005, *n* = 18387). The outcome variables were register-based, and the follow-up was from 2006 until 2015. The risk estimates were evaluated using competing risk survival analysis.

**Results:**

There were 530 (3%) disability pension events due to musculoskeletal disorders during the 10-year follow-up. After adjustment for sex, age, education and chronic diseases, employees in the second (SHR = 1.46, 95% CI 1.05–2.05), third (SHR = 2.73, 95% CI 2.00–2.29), and the highest exposure quartile (SHR = 2.56, 95% CI 1.88–3.50) had a higher risk of musculoskeletal disability pension than employees in the lowest quartile. A total of 110 (4%) men and 266 (2%) women died during the follow-up. Men in the third quartile (SHR = 2.29, 95% CI 1.23–4.24), and women in the highest exposure quartile (SHR = 1.54, 95% CI 0.99–2.41) had a higher risk of premature mortality than those in the lowest quartile.

**Conclusions:**

Eight to ten years of exposure to heavy physical effort at work is strongly associated with disability pension due to musculoskeletal disorders. This exposure also increases the risk of premature mortality, particularly among men.

**Electronic supplementary material:**

The online version of this article (10.1007/s00420-018-1393-5) contains supplementary material, which is available to authorized users.

## Introduction

Physically demanding work has been widely shown to be a risk factor for disability pension (Karpansalo et al. 2002; Krokstad et al. 2002; Labriola et al. 2009; Lahelma et al. 2012), and is linked specifically to a higher risk of disability pension due to musculoskeletal disorders (Karkkainen et al. 2013; Lahelma et al. 2012). However, less is known about how long-time continuous exposure to physically demanding work affects the risk of disability pension. Two studies, one among Finnish twins (Ropponen et al. 2014), and another among two Swedish birth cohorts (Kjellberg et al. 2016), showed that long-term exposure to physically demanding work was associated with a higher risk of disability pension due to musculoskeletal disorders. However, the measure of long-term exposure was not based on annual follow-up of exposure, but on two separate measurement points 5–6 years apart without information on potential changes in exposure between the measurements. A recent study with Danish register cohort data found that lifting-years, but not kneeling- or vibration-years, were associated with an increased risk of all-cause disability pension (Sundstrup et al. 2017). However, the study did not separately examine musculoskeletal disorder-related disability pensions.

Some of the studies on the association between physical workload and disability pension have used exposure estimates derived from job exposure matrices (JEM) that evaluate job exposures based on occupational titles. Each occupation receives and exposure estimate based on survey responses, face-to-face interviews, or on expert evaluations (Kjellberg et al. 2016; Solovieva et al. 2012; Sundstrup et al. 2017). Particularly for physical work exposures, these matrices have shown rather high specificity and sensitivity, and validation studies support their use when individual exposures are unavailable (Dale et al. 2015; Rijs et al. 2014; Solovieva et al. 2012). However, the exposure values (which originally are percentages, i.e., continuous variable) are often dichotomized or otherwise grouped. This decision dilutes variation, and information is lost. Hence, in this study, we kept the annual job exposure estimates as percentages, and calculated the average exposure during the exposure follow-up.

In addition to disability pension, heavy physical work may increase the risk of premature death, i.e., death during working age, particularly among men (Holtermann et al. 2009, 2010a, 2012). Thus, death should be viewed as competing risk when evaluating the extent to which heavy physical work increases the risk of disability pension. The aim of this study was to develop a new JEM based on the Helsinki Health Study (HHS) survey data to assess long-term exposures to physically demanding work. The HHS–JEM was then used to build a prognostic model to estimate the sub-distribution hazards of disability pension due to musculoskeletal disorders and death among people with different exposures to physically heavy work (Austin and Fine 2017; Putter et al. 2007). We used a competing risk model to examine whether long-term exposure to heavy physical effort estimated with JEM was associated with disability pension due to musculoskeletal disorders and all-cause premature mortality.

## Methods

### Compilation of the job exposure matrix (HHS–JEM)

*JEM population* We used data from the Helsinki Health Study (HHS), which focuses on health and working conditions of employees of the City of Helsinki, Finland. The baseline data were derived from questionnaire surveys conducted in 2000–2002 including employees reaching the age of 40, 45, 50, 55 or 60 years each year. In total, 8960 employees (80% women) responded at baseline with the response rate of 67%. The HHS protocol has been approved by ethics committees of the health authorities of the City of Helsinki and the Department of Public Health, University of Helsinki, Finland (Lahelma et al. 2013).

*Exposure information* In the HHS survey, the respondents reported whether heavy physical effort or heavy lifting and carrying were present in their work. The scale was: 0 (does not occur); 1 (occurs, but does not bother); 2 (occurs, and bothers to a moderate degree); 3 (occurs and bothers to a large degree). Value 0 was categorized as “unexposed”, and all the other values as “exposed”. The JEM estimate was calculated as the prevalence of exposure (as percentage) in each occupational title.

*Occupational classification* Occupational titles based on employer’s register data were transformed to match occupational titles based on coding of Statistics Finland (Tilastokeskus 1999). The coding of occupations varies from one to three or four digits. More digits indicate finer classification: code ‘0’ groups all technical, scientific, legal, humanistic, and artistic work; ‘00’ groups all technical work; ‘001’ groups all architects. We could match an occupational title for 6789 baseline survey respondents. A total of 132 different occupational titles were found (3-digit classification). As there were 218 (3-digit) occupational titles in the codebook of Statistics Finland, our HHS survey respondents represented 61% of all occupational titles in municipal work.

*The HHS–JEM* The JEM estimates were calculated separately for men (*n* = 1381) and women (*n* = 5378). Small occupational groups were merged into larger (2 digit) classifications, if two experts (JE and MM) agreed in favor of merging the occupations. Before occupational titles were merged, we checked that the JEM estimates were similar enough for the two groups. We omitted 66 (50%) occupational titles with few respondents and which could not be merged due to differential work tasks and occupational exposure. In the final HHS–JEM, there were 40 occupational titles (13 with 2-digit classification, and 27 with 3-digit classification), which covered 30% of all occupational titles in municipal work. When considering only those occupations that HHS survey respondent held, the HHS–JEM covered 50% of occupations. Men had JEM estimates for 21 occupational titles, which were based on responses of 1168 men (65% of all male respondents). Women had JEM values for 36 occupational titles, which were based on responses of 5189 women (72% of all female respondents). In total, the HHS–JEM covered 71% of all survey respondents in 2000–2002. The minimum number of respondents for whom the JEM estimates were calculated was 18 for men, and 24 for women. The occupational titles and number of respondents are detailed in Online Resource 1.

### Study population

**Fig. 1:**
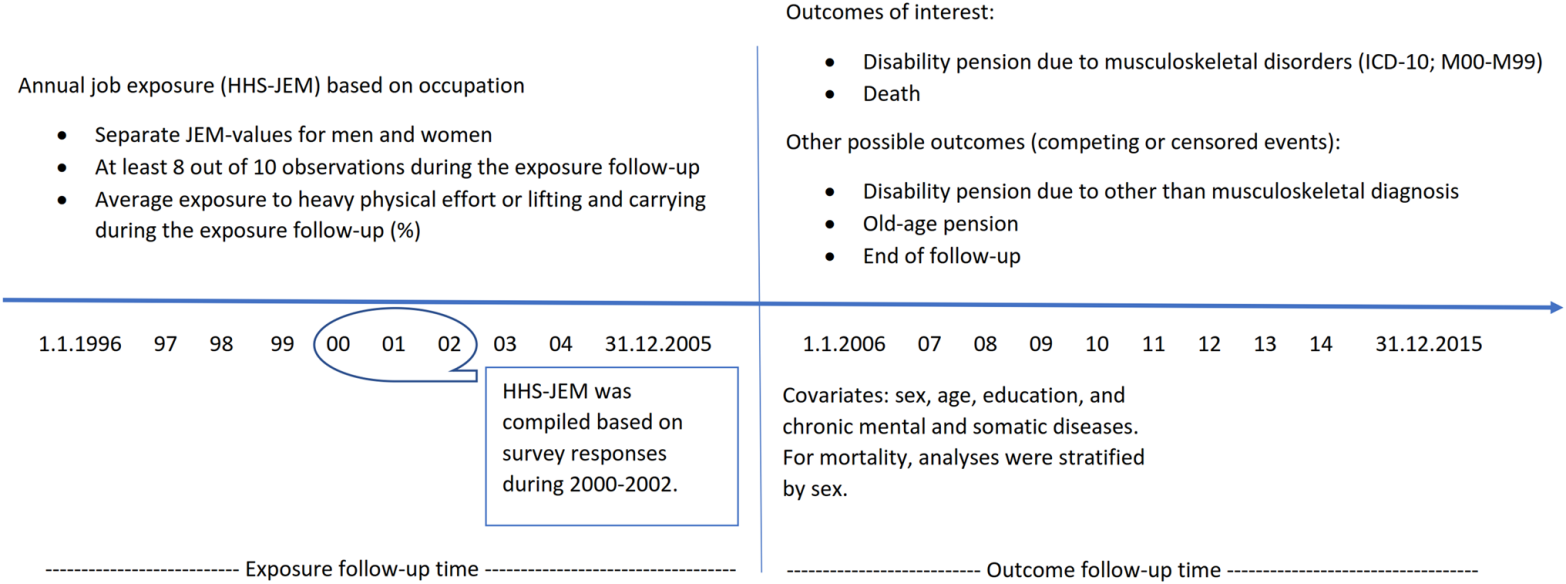
Study design, variables and follow-up. HHS–JEM = job exposure matrix based on the Helsinki Health Study

The study population were all employees of the City of Helsinki between 1996 and 2005 (*n* = 118,122). For this cohort, we had employer’s register data, including employees’ occupational titles. We linked the HHS–JEM data for the City of Helsinki employee cohort based on their occupational titles. Using national personal identification numbers, we were also able to link register data from the Finnish Centre for Pensions covering all granted pensions based on disability including ICD-10 coded diagnoses (International Statistical Classification of Diseases and Related Health Problems (ICD-10) 1994), as well as old age pensions. In addition, we had information on age, sex, education, and mortality for all causes from the Statistics Finland. Information on prescription medication purchases and special medication reimbursements were from the Social Insurance Institution of Finland. Information regarding notifications of diagnosed malignant tumors were from the Institute of Statistical and Epidemiological Cancer Research (the Finnish Cancer Registry). The linkage of the JEM estimate (at least for one out of 10 years) succeeded for a total of 98,834 employees. From this cohort, the eligible population were those who were alive and not on disability or old age pension before 1st January 2006 (*n* = 87,130). We omitted employees with less than 8 years of JEM estimates, and those without information on covariates resulting in 18,387 employees for our final analytical sample. Of the employees 84% were women reflecting the sex distribution in the municipal sector. The study design is further described in Fig. 1.

### Exposure

We followed occupational exposure to heavy physical effort for 10 years, from January 1, 1996 until December 31, 2005. Those with less than 8 observation-years during the exposure follow-up were excluded. The annual exposure was computed into the mean exposure during the exposure follow-up time (percentage). For most participants (64%), their occupational title did not change (i.e., the exposure was on the same level throughout the exposure follow-up). For 36%, there were changes in exposure during the follow-up. The mean exposure variable was classified into quartiles as follows: 0–25.9%, 26–64.9%, 65–82.6%, ≥ 82.7%. In sensitivity analyses, we used continuous exposure variable.

### Outcomes

In Finland, a person who is unable to work is eligible to receive a sickness absence benefit for a maximum of 1 year. After that, if the person’s work ability remains reduced at least by 60% (remains unable to work), a full disability pension can be granted (either for a fixed period or as a permanent disability pension). The decision on pension is based on physician certificate and a medical diagnosis and is made by an insurance institution. If part-time work is possible, part-time disability pension can be granted when work ability is reduced at least by 40%. We obtained register-based information on the dates of granted disability pensions from the Finnish Centre for Pensions (data on temporary, permanent, full-time and part-time disability pensions) coded according to the ICD-10 (International Statistical Classification of Diseases and Related Health Problems (ICD-10) 1994). We examined disability pension due to musculoskeletal disorders (M00-M99), which cover about 30% of all granted pensions (Statistical yearbook of pensioners in Finland 2016 2017).

Information on mortality (date of death) was retrieved from Statistics Finland register of causes of death. The outcome follow-up was 10 years, from January 1, 2006 until December 31, 2015. We defined death and disability pension due to other than musculoskeletal diagnosis as competing event to disability pension due to musculoskeletal disorders. However, in the mortality analyses, disability pension was not regarded as competing event to death.

### Covariates

Age and educational level were obtained from Statistics Finland. Educational level was classified into 0 (secondary education or less); 1 (tertiary education, undergraduate); 2 (tertiary education, graduate/doctoral degree). Chronic somatic conditions were: cancer (diagnosed during 2003–2005 from the Cancer Registry), and diabetes, cardiac failure, coronary artery disease, stage 2 hypertension, rheumatoid arthritis, asthma, Parkinson’s disease, epilepsy, uremia, bowel disease, multiple sclerosis, and diseases of pancreas as defined through special medication reimbursement valid at the start of the outcome follow-up on 1st January 2006. In addition, we defined mental disorders from medication purchases with the Anatomical Therapeutic Chemical Classification (ATC) codes N05 (psycholeptics) and N06 (psychoanaleptics) during 2003–2005. The presence of chronic disease was defined as having at least one of these proxies for somatic or mental condition.

### Statistical analysis

For general description of the data, we used frequency tables, means and standard deviations. We tested effect modification by including interaction term ‘sex × exposure’ into Cox proportional hazards models (time to disability pension and time to death). As we observed statistically significant (*p* < 0.001) interaction regarding mortality, those analyses were stratified by sex.

We used Kaplan–Meier estimator method to estimate survival functions to compare time to disability pension and death for the exposed and the unexposed, and to visually evaluate the assumption of proportional hazards. We tested the proportional hazards assumption by including an interaction of the exposure with the log of follow-up time. The interaction terms were non-significant (*p* values > 0.05) justifying the proportional hazards assumption.

We used Cox proportional hazards models to examine the association between exposure to heavy physical work and incident mortality. The interaction test indicated effect modification by sex, and the analyses were stratified by sex. The results were presented as subhazard ratios (SHR) and their 95% confidence intervals (CI). The follow-up was until death or the end of the follow-up (December 31, 2015), whichever came first.

For disability pension due to musculoskeletal disorders, we used Cox proportional hazards model, but with death and disability pensions due to other than M00–M99 diagnoses were treated as competing risks. Cases were censored in the event of old-age pension, or when reaching the age of 63 after which disability pension can no longer be granted, or at the end of the follow-up. Compared to the standard survival analysis, where the follow-up of non-events terminates only due to censoring, competing risk analysis considers competing events that prevent the event of interest from occurring. Treating observations that experience competing events as if they could later experience the event of interest overestimates the probability of failure, and the bias is larger when the competition due to frequent competing events is heavier (Putter et al. 2007).

We tested whether the association was linear or non-linear by including each exposure squared in addition to exposure treated as a linear term. The results mainly supported non-linear association. Thus, we show the results with classified exposure variable (supporting non-linear association) as main results, and as continuous variable (supporting linear association) in sensitivity analysis (Online Resource 2).

## Results

Of the participants (*n* = 18,387), 84%, were women. The characteristics of the participants at the beginning of the outcome follow-up period are described in Table 1 stratified by sex. Men were slightly older and had higher level of education than women. No difference was observed for chronic disease prevalence between sexes. Women had more exposure to heavy physical effort or lifting and carrying than men.

**Table 1:** Characteristics of study participants by sex (*n* = 18,387)

	Men (*n* = 2870)			Women (*n* = 15,517)		
	%	Mean	SD	%	Mean	SD
Secondary education or lower	30			43		
Tertiary education/undergraduate	35			37		
Tertiary education/graduate	35			20		
Chronic disease (at the beginning of the follow-up)	24			25		
Age (at the beginning of the follow-up)		48.3	8.3		47.0	8.5
Exposure to heavy physical work/lifting and carrying (during 1996–2005)		39.7	27.8		54.9	26.8

### Disability pension due to musculoskeletal disorders

**Fig. 2:**
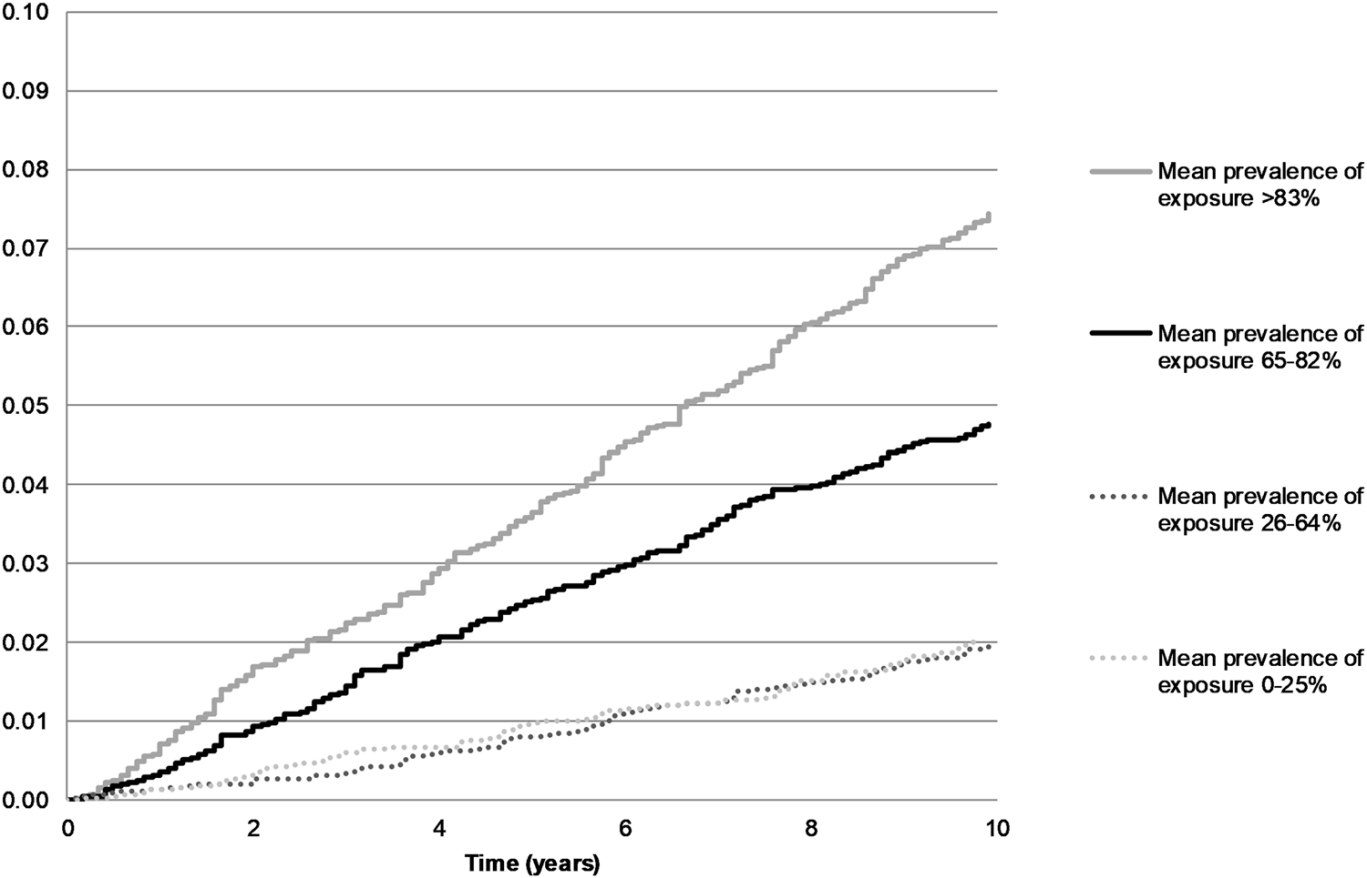
Cumulative probability of disability pension due to musculoskeletal disorders stratified by the average level of 8–10 years of continuous exposure to heavy physical effort at work

Figure 2 shows Kaplan–Meier curves for time to disability pension due to musculoskeletal disorders stratified by the average exposure to heavy physical effort. A total of 5–7% of employees with above median (3rd and 4th quartile) exposure to heavy physical work ended up on musculoskeletal disorder-related disability pension during the 10-year follow-up, whereas the corresponding percentage for those with the lowest level of exposure was 2%.

The mean follow-up time for disability pension due to musculoskeletal disorders was 8.5 years (SD 2.7). There were 530 (3%) musculoskeletal disability pension events during the follow-up. Compared to employees in the lowest quartile of average level of exposure to heavy physical effort, employees in all other exposure quartiles had a higher risk of disability pension due to musculoskeletal disorders (Table 2). The analysis with the linear exposure variable showed a 1.15 (95% CI 1.10–1.20) times higher risk of musculoskeletal disability pension for a 10%-unit increase in average exposure to heavy physical effort (Online Resource 2.)

**Table 2:** 8–10 years of exposure to heavy physical effort at work and status at the end of follow-up (*n* = 18,387)

	Disability pension due to musculoskeletal disorder^a^		Death^a,b^			
	530 events (3%)		Men, 110 events (4%)		Women, 266 (2%)	
	SHR^c^	95% CI	SHR	95% CI	SHR	95% CI
Lowest quartile	1		1		1	
2nd quartile	1.46	1.05–2.05	0.87	0.52–1.46	1.24	0.87–1.75
3rd quartile	2.73	2.00-3.72	2.29	1.23–4.24	1.16	0.79–1.71
Highest quartile	2.56	1.88–3.50	1.70	0.90–3.20	1.54	0.99–2.41

^a^Adjusted for age, educational level, chronic disease and sex

^b^The interaction with sex was statistically significant (*p* < 0.001), and the analyses were stratified by sex and adjusted for age, educational level, and chronic diseases

^c^530 events of interest, 816 competing events, 17,034 censored

As a supplementary analysis, we further stratified the outcome by sub-blocks of musculoskeletal diagnoses (Online Resources 3 and 4). Above median (3rd and 4th quartile) exposure to heavy physical effort showed approximately threefold risk for arthropathies, specifically for arthrosis (four–fivefold risk). The risks for dorsopathies and soft tissue disorders were also significantly elevated for those with above median exposure (Online Resource 4).

### Risk of premature death

**Fig. 3:**
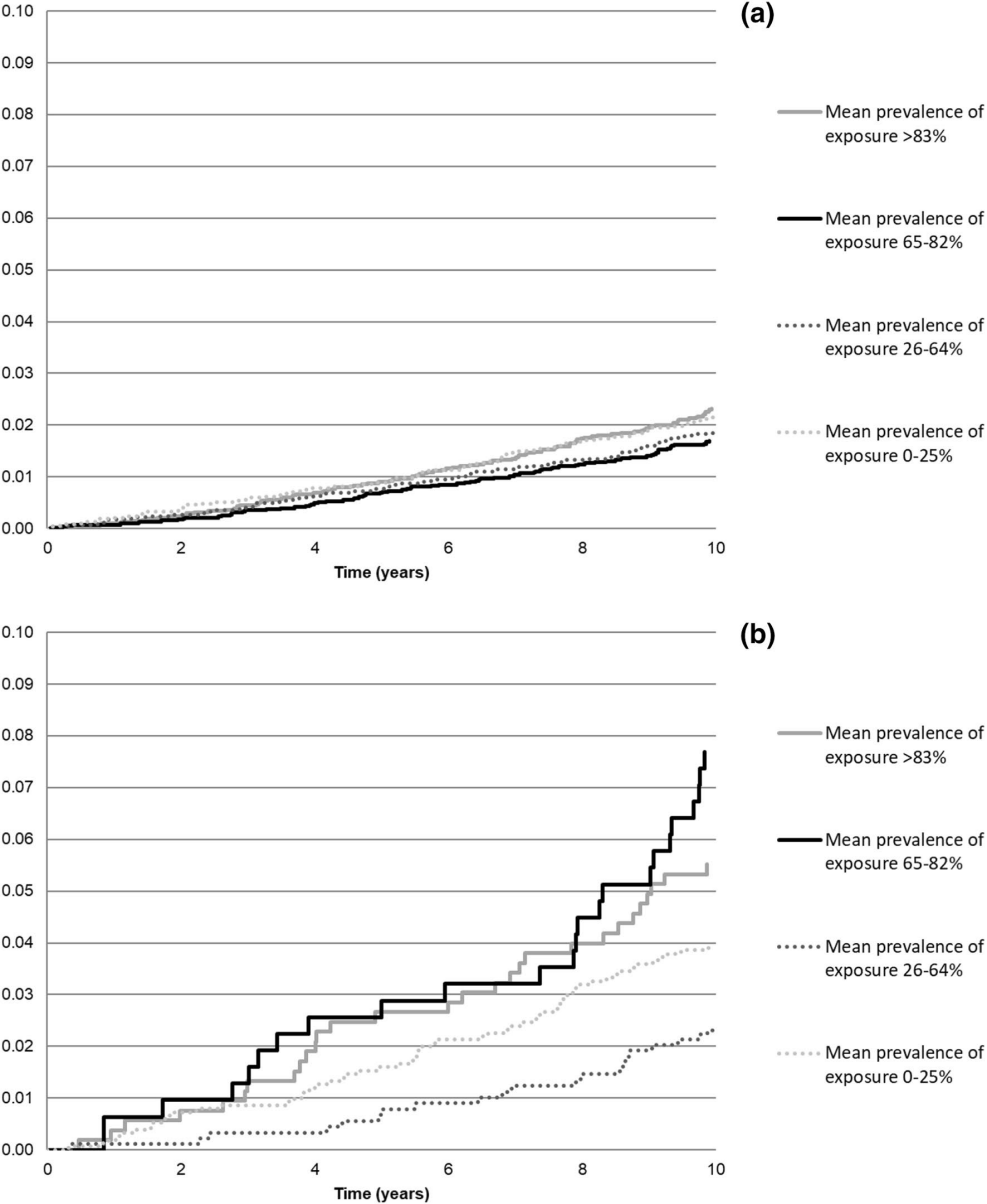
Cumulative probability of premature mortality stratified by the average level of 8–10 years of continuous exposure to heavy physical effort at work among **a** women and **b** men

Figure 3 (Panel a for women and panel b for men) shows Kaplan–Meier survival curves stratified by average exposure to heavy physical effort. Among women, no difference in premature mortality was observed by exposure to heavy physical effort. Around 1.6–2.3% of women died during the 10-year follow-up. Among men, a higher overall death rate was observed. A total of 5–8% of men with above median (3rd and 4th quartile) exposure to heavy physical work died during the 10-year follow-up, whereas the corresponding percentage with below median exposure was from 2 to 4%.

The mean follow-up time for premature death was 9.9 years (SD 0.7). There were 266 (2%) deaths among women and 110 (4%) among men during the 10-year follow-up. Among men, the second highest exposure (65–82%) to heavy physical effort was associated with a higher risk of premature mortality (SHR = 2.29, 95% CI 1.24–4.26) compared to employees in the lowest quartile of average level of exposure (Table 2). The higher risk of premature mortality among men was also observed when using dichotomized exposure variable (median split): the hazard ratio was 2.11 (95% CI 1.27–3.49) among men who had above median exposure to heavy physical effort compared to those with below median level of exposure (data not shown in Tables). An analysis with linear exposure variable showed a 1.13 (95% CI 1.04–1.22) times higher risk of premature mortality for a 10%-unit increase in average exposure to heavy physical effort (Online Resource 2).

Among women, the highest exposure (> 82%) to heavy physical effort was marginally associated with higher risk of premature mortality (SHR = 1.54, 95% CI 0.99–2.41) (Table 2). The SHR was 1.03 (0.98–1.09) for 10%-unit increase in average exposure in the analysis with the linear exposure variable in women (Online Resource 2).

## Discussion

We compiled JEM estimates for various municipal sector occupations regarding exposure to heavy physical effort or heavy lifting and carrying. As a primary validation of the JEM, the associations with disability pension due to musculoskeletal disorders were examined. Confirming our hypothesis, the study demonstrated an association between 8–10 years of continuous exposure to heavy physical effort and disability pension due to musculoskeletal disorders. In addition, we observed an increased risk of premature death particularly among working age men who had 8–10 years of exposure to heavy physical effort or lifting and carrying.

Our results correspond to previous studies, which supports the validity of the municipal sector JEM (Karkkainen et al. 2013; Karpansalo et al. 2002; Kjellberg et al. 2016; Krokstad et al. 2002; Labriola et al. 2009; Lahelma et al. 2012; Ropponen et al. 2014; Sundstrup et al. 2017). However, we also add to the existing literature, because we measured the physical exposures continuously during 8–10 years prior to the outcome follow-up period. The duration of the exposure (i.e., how long participants have performed physically demanding tasks) has not been examined in previous studies. In this study, we could first follow the exposure for 10 years, and then follow the outcome for 10 more years. The risk for musculoskeletal disability pension was strongly increased with 8–10 years of continuous exposure to physically demanding work.

Moreover, we examined the relationship between 8–10 years of exposure to heavy physical effort and premature mortality in working age. Congruent to previous studies (Holtermann et al. 2009, 2010a, b, 2012), men with heavy physical work had an elevated risk of premature death. The association between exposure to heavy physical effort and mortality was significant among those with second highest (third quartile) exposure, but not among those with the highest exposure. This may imply health selection, where only very healthy men in physically demanding occupations continue to work for 8–10 years. We expanded earlier research as we also studied this association among women, and found a significant association in the highest exposure quartile.

### Strengths and weaknesses

A major strength of the current study was the 20-year time frame, where we first followed the exposure for 10 years, and then the outcome for another 10 years. JEM was based on physical work exposure during 2000–2002. We generalized the occupational exposures of 2000–2002 to 4 years before and 3 years after (i.e., to 1996–2005) assuming that no major changes in work and working conditions affecting physical exposures in these occupations occurred during that time. Moreover, we kept the JEM estimates as continuous percentage variables (0–100%), and calculated the mean level of exposure during 8–10 years for each participant. With this approach, we could ascertain the duration of exposure, in addition to include information about the variation in intensity of exposure, should there have been a change in occupation and subsequent level of physical exposure. However, to obtain a measure of long-term continuous exposure, we had to omit all participants with less than 8 years of information on occupation and subsequent occupational exposures. This may have resulted in selection of fitter and healthier workers who remained employed with the City of Helsinki (i.e., did not resign, die, or end up in disability pension until 31 December 2005), which may have led to underestimation of the effect of exposure to physically demanding work on disability pension and mortality. As our exposure estimate was based on occupational title, we were unable to account for work modification to less physically demanding within the same occupational title. Nevertheless, a change into less strenuous occupational title during the 10-year exposure follow-up was accounted for.

In most previous studies, the analysis of the risk of disability pension has not considered competing events. We treated death and disability pension with other than musculoskeletal diagnosis as competing events to disability pension due to musculoskeletal disorders.

Measuring heavy physical effort at work with JEM can be considered as a strength and as a weakness of the study. On one hand, aggregate measures are not able to tap within-job and individual variance in physical demands. On the other hand, previous JEM validation studies have found that these matrices have rather good validity, sensitivity and specificity, particularly regarding physical exposures (Rijs et al. 2014; Solovieva et al. 2012). Due to small number of respondents in some occupational titles, we had to omit about 50% of occupations from further analysis. This may have implication to generalizability. However, selecting only occupations with enough respondents improves the validity of our measures, as they are not based on evaluations of few respondents. Moreover, aggregation of self-reported exposure data decreases the effects of recall bias and individual characteristics including mood, personality, and health status, which may influence individual appraisals of the exposure.

We considered confounding through sex, age, education, and chronic diseases, which were available from registers. We did not have information on health behaviors, including physical fitness, body mass index, alcohol use, and smoking. In an earlier study, the effect of physical risk factors on disability pension was robust to adjustment for health behaviors (Karpansalo et al. 2002). In some previous studies, high levels of physical fitness have been observed to protect from the adverse effects high physical work demands on premature mortality (Holtermann et al. 2010a, 2012). However, it is very difficult to disentangle the effects of heavy physical effort at work from other risk factors that also pertain among men doing heavy manual work, for example, high blood pressure and smoking. Exposure to heavy physical work may include other hazardous exposures as well (for example, exposure to asbestos) or built-in socially structured inequality in health. This kind of mixed exposure to physical, ergonomic, or psychosocial factors can produce health consequences that are additive or synergistic (Mixed Exposures Research Agenda. A Report by the NORA Mixed Exposures Team 2004), and may explain the observed increased risk of mortality.

We did not adjust for psychosocial work environment factors. Occupation-specific aggregate measures of psychosocial exposures, including psychological demands or social support (i.e., psychosocial JEM), have shown poorer validity than physical exposures (Niedhammer et al. 2008; Rijs et al. 2014; Solovieva et al. 2014). This is plausible, since the level of, for example, coworker support may not be as dependent from one’s occupation as it is from one’s work unit, and even from one’s individual resources and the fit between person and the work environment. Hence, we did not compile a psychosocial JEM. Moreover, earlier studies have shown that the effect of physical exposure is robust to adjustment for psychosocial factors (Friis et al. 2008; Kjellberg et al. 2016; Labriola et al. 2009; Lahelma et al. 2012).

## Conclusion

Eight to ten years of continuous exposure to heavy physical effort or heavy lifting and carrying at work was associated with an increased risk of disability pension due to musculoskeletal disorders. Furthermore, continuous exposure to heavy physical effort or lifting and carrying was associated with premature mortality, particularly among men. In addition to physically demanding work causing musculoskeletal disorders and injuries, these jobs may also have lower possibilities to adjust work to accommodate reduced health and functioning. Effective ways to accommodate physically demanding work environment and work tasks to meet the reduced physical functioning are therefore needed.

## Electronic supplementary material

Below is the link to the electronic supplementary material.

Supplementary material 1 (DOCX 22 KB)

Supplementary material 2 (DOCX 13 KB)

Supplementary material 3 (DOCX 14 KB)

Supplementary material 4 (DOCX 14 KB)
