# Supplementary material for: Long-term exposure to heavy physical work, disability pension due to musculoskeletal disorders and all-cause mortality: 20-year follow-up—introducing Helsinki Health Study job exposure matrix
Source: Int Arch Occup Environ Health. 2018 Dec 3;92(3):337–45. doi: 10.1007/s00420-018-1393-5 (PMC6420465; doi:10.1007/s00420-018-1393-5)
Supplement: Supplementary file 1 — Supplementary material 1 (DOCX 22 KB) [file 420_2018_1393_MOESM1_ESM.docx]

**Online Resource 1.** Description of included occupational titles, number of male and female respondents in each occupational title category, and JEM-values (i.e., percentage reporting heavy physical effort or lifting and carrying at work).

| Occupational title, 2-digit code | Occupational title, 3-digit code | Code | Notes | n of men | JEM-value | n of women | JEM-value |
| --- | --- | --- | --- | --- | --- | --- | --- |
| Technical work |  | 00 | architects, engineers, technicians, mappers, draughtsman, other technical work | 231 | 20.52 | 86 | 12.79 |
| Teachers | Secondary vocational education | 032 |  | 60 | 21.67 | 132 | 17.83 |
|  | Pre-school | 033 |  | 4* |  | 257 | 71.37 |
|  | Basic education |  |  | 88 | 40.91 | 317 | 35.03 |
|  | Lecturer in basic or secondary schools | 034 |  | 38 | 23.68 | 113 | 34.51 |
|  | Special education | 035 |  | 4* |  | 28 | 37.04 |
|  | Adult education | 038 |  | 39 | 15.38 | 134 | 29.77 |
|  | Other education | 039 |  | 8* |  | 60 | 28.07 |
| Writing work, journalistic work |  | 06 | journalists, writers, program editors, public relations officers, translators | 5* |  | 32 | 12.5 |
| Library, archiving, museum work |  | 08 | librarians, archivists, recorders, museum officers | 18 | 61.11 | 76 | 56 |
| Health care work | Medical doctors | 101 |  | 43 | 9.3 | 65 | 14.06 |
|  | Chief nursing officers | 102 |  | 0* |  | 27 | 0 |
|  | Nursing staff | 103 |  | 30 | 80 | 764 | 68.21 |
|  | Other health care work | 104 |  | 6* |  | 115 | 82.61 |
| Therapy work |  | 11 | physical therapists, occupational therapists | 0* |  | 31 | 61.29 |
| Dental care work | Dentists | 121 |  | 4* |  | 37 | 13.51 |
|  | Dental nurses | 122 |  | 0* |  | 60 | 27.12 |
| Social work | Social workers in managerial or administrative work | 151 |  | 20 | 30 | 172 | 34.12 |
|  | Specialized social workers | 152 |  | 12* |  | 171 | 12.87 |
|  | Child care (public day care) | 154 |  | 3* |  | 488 | 83.65 |
|  | Child care (family day care/private childminders) | 155 |  | 0* |  | 186 | 84.86 |
|  | Craft leaders, hobby leaders | 156 |  | 34 | 64.71 | 106 | 70.75 |
|  | Home aids, home helps | 157 |  | 4* |  | 242 | 91.98 |
|  | Other social work | 159 |  | 18 | 88.89 | 185 | 88.2 |
| Environment and health protection |  | 16 | health officers, occupational safety officers | 19 | 15.79 | 10* |  |
| Psychologists and speech therapists |  | 17 | speech therapists (code 113) were omitted from therapy work and combined with psychologist due to more similar occupational exposures | 5* |  | 60 | 8.47 |
| Youth work, sports, and exercise work |  | 18 | youth workers, sport leaders, physical education instructors, recreational work | 8* |  | 24 | 60.87 |
| Managerial-level clerical work, other societal-administrative work |  | 20 | chief officers, local government administers, presenting officials, reporting officials, other senior managers, finance directors | 167 | 10.84 | 184 | 8.7 |
| Personnel administration, personnel management |  | 22 | employment agency officers were omitted due to their differential job exposures | 3* |  | 63 | 12.7 |
| Financial and accounting work |  | 23 | auditors, accountants, bookkeepers, cashiers (in offices) | 3* |  | 38 | 13.16 |
| Secretarial work, assisting office work |  | 24 | office secretaries, clerks, calculation of pay | 39 | 57.89 | 433 | 22.12 |
| Automatic data processing work |  | 25 | Chief ADP officers, ADP programmers, operators | 60 | 28.33 | 26 | 28 |
| Traffic work | Public transportation work | 641 | Motor vehicle drivers,  tram drivers, engine drivers, metro engine drivers | 146 | 12.41 | 27 | 20 |
| Other transportation and traffic work |  | 69 | lighthouse guards, harbor guards, other transportation and traffic work | 20 | 65 | 3* |  |
| Protection work | Firemen | 901 | firemen | 50 | 91.67 | 0* |  |
| Hotel, restaurant, institutional catering work | Chefs, cooks | 912 | chefs, cooks | 3* |  | 112 | 90.91 |
|  | Kitchen help | 913 | kitchen help | 3* |  | 69 | 93.65 |
| Janitorial service and cleaning work | Janitorial service and real estate work | 931 | janitors, real estate managers | 48 | 86.96 | 13 |  |
|  | Cleaning work | 932 | cleaners, managers in cleaning work | 2* |  | 124 | 85.83 |
|  | Other janitorial and cleaning work | 939 | other janitorial and cleaning work | 7* |  | 145 | 89.29 |

* JEM not calculated due to low number of respondents
