# Supplementary material for: Long-term exposure to heavy physical work, disability pension due to musculoskeletal disorders and all-cause mortality: 20-year follow-up—introducing Helsinki Health Study job exposure matrix
Source: Int Arch Occup Environ Health. 2018 Dec 3;92(3):337–45. doi: 10.1007/s00420-018-1393-5 (PMC6420465; doi:10.1007/s00420-018-1393-5)
Supplement: Supplementary file 2 — Supplementary material 2 (DOCX 13 KB) [file 420_2018_1393_MOESM2_ESM.docx]

**Online Resource 2.** Long-term exposure to heavy physical effort/lifting and carrying. Exposure variables as continuous (Subdistribution hazards per 10% increase in exposure).

|  | SHR^a^ | 95% CI | SHR^b^ | 95% CI |
| --- | --- | --- | --- | --- |
| Disability pension due to musculoskeletal disorders | 1.25 | 1.21-1.29 | 1.15 | 1.10-1.20 |
| Premature mortality: Men | 1.08 | 1.02-1.14 | 1.13 | 1.04-1.22 |
| Premature mortality: Women | 1.00 | 0.96-1.04 | 1.03 | 0.98-1.09 |

^a^ Unadjusted

^b^ Adjusted for sex, age, education, and chronic disease (except for mortality, where the analyses were stratified by sex).
