# Supplementary material for: Long-term exposure to heavy physical work, disability pension due to musculoskeletal disorders and all-cause mortality: 20-year follow-up—introducing Helsinki Health Study job exposure matrix
Source: Int Arch Occup Environ Health. 2018 Dec 3;92(3):337–45. doi: 10.1007/s00420-018-1393-5 (PMC6420465; doi:10.1007/s00420-018-1393-5)
Supplement: Supplementary file 3 — Supplementary material 3 (DOCX 14 KB) [file 420_2018_1393_MOESM3_ESM.docx]

**Online Resource 3.** Cases of disability pension due to musculoskeletal disorder diagnoses by exposure to heavy physical effort or lifting and carrying at work (quantiles). Frequency (percentage).

|  | **Lowest exposure** | **2^nd^** | **3^rd^** | **Highest exposure** | **P for difference** |
| --- | --- | --- | --- | --- | --- |
| M00-M99: All musculoskeletal | 60 (11) | 81 (15) | 178 (34) | 211 (40) | <0.001 |
| M00-M25: Arthropathies | 27 (11) | 36 (14) | 77 (30) | 115 (45) | <0.001 |
| M15-M19: arthrosis | 19 (9) | 29 (13) | 68 (31) | 101 (47) | <0.001 |
| M30-M36: Systemic connective tissue | 5 (50) | 1 (10) | 2 (20) | 2 (20) | 0.27 |
| M40-M54: Dorsopathies | 24 (12) | 34 (16) | 76 (37) | 73 (35) | <0.001 |
| M50-M54: other dorsopathies | 17 (14) | 20 (16) | 46 (38) | 39 (32) | <0.001 |
| M60-M79: Soft tissue | 2 (5) | 7 (16) | 18 (42) | 16 (37) | <0.001 |
| M70-M79: other soft tissue | 2 (5) | 7 (17) | 17 (41) | 15 (37) | 0.001 |
| M80-M94: Osteopathies and chondropathies | 2 (17) | 1 (8) | 4 (33) | 5 (42) | 0.21 |
