# Supplementary material for: Long-term exposure to heavy physical work, disability pension due to musculoskeletal disorders and all-cause mortality: 20-year follow-up—introducing Helsinki Health Study job exposure matrix
Source: Int Arch Occup Environ Health. 2018 Dec 3;92(3):337–45. doi: 10.1007/s00420-018-1393-5 (PMC6420465; doi:10.1007/s00420-018-1393-5)
Supplement: Supplementary file 4 — Supplementary material 4 (DOCX 14 KB) [file 420_2018_1393_MOESM4_ESM.docx]

**Online Resource 4**. 8-10 years of exposure to heavy physical effort at work (by quantiles) and risk of disability pension due to musculoskeletal diagnoses. SHR=subhazard ratio adjusted for sex, age, education, and chronic disease. 95% CI = 95% confidence interval.

|  | Lowest | 2^nd^ |  | 3^rd^ |  | Highest |  |
| --- | --- | --- | --- | --- | --- | --- | --- |
|  | SHR | SHR | 95% CI | SHR | 95% CI | SHR | 95% CI |
| M00-M25: Arthropathies | 1 | 1.48 | 0.89-2.44 | 2.64 | 1.66-4.20 | 3.36 | 2.12-5.32 |
| M15-M19: arthrosis | 1 | 1.75 | 0-98-3.11 | 3.63 | 2.12-6.21 | 4.72 | 2.75-8.13 |
| M40-M54: Dorsopathies | 1 | 1.50 | 0.88-2.56 | 2.84 | 1.74-4.63 | 2.05 | 1.24-3.38 |
| M50-M54: other dorsopathies | 1 | 1.18 | 0.61-2.29 | 2.04 | 1.13-3.68 | 1.33 | 0.73-2.44 |
| M60-M79: Soft tissue | 1 | 3.58 | 0.80-16.1 | 7.56 | 1.80-31.8 | 4.34 | 1.12-16.8 |
| M70-M79: other soft tissue | 1 | 3.65 | 0.81-16.3 | 7.37 | 1.73-31.3 | 4.23 | 1.08-16.6 |
